# Impact of Tumor Necrosis Factor Antagonist Therapy on Circulating Angiopoietin-like Protein 8 (ANGPTL8) Levels in Crohn’s Disease—A Prospective Multi-Center Study

**DOI:** 10.3390/jcm14145006

**Published:** 2025-07-15

**Authors:** Mohammad Shehab, Sharifa Al-Fajri, Ahmed Alanqar, Mohammad Alborom, Fatema Alrashed, Fatemah Alshammaa, Ahmad Alfadhli, Sriraman Devarajan, Irina Alkhairi, Preethi Cherian, Jehad Abubaker, Mohamed Abu-Farha, Fahd Al-Mulla

**Affiliations:** 1 Division of Gastroenterology, Department of Internal Medicine, Mubarak Alkabeer, University Hospital, Kuwait University, Jabriya 47060, Kuwait; smohammadalfajri@moh.gov.kw (S.A.-F.); mohammad.alborom@ku.edu.kw (M.A.); ahalshammaa2979@moh.gov.kw (F.A.); ahalfadhli@moh.gov.kw (A.A.); 2 Department of Translational Research, Dasman Diabetes Institute, Dasman, Kuwait City 15462, Kuwait; 3 School of Medicine Building, Faculty of Medicine, Royal College of Surgeons in Ireland, Busaiteen 15503, Bahrain; ahmedalanqar2023@alumnircsi.com; 4 Department of Pharmacy Practice, Faculty of Pharmacy, Kuwait University, Jabriya 13110, Kuwait; fatema.alrashed@ku.edu.kw; 5 Department of Biochemistry and Molecular Biology, Dasman Diabetes Institute, Dasman, Kuwait City 15462, Kuwait; sriraman.devarajan@dasmaninstitute.org (S.D.); irina.alkhairi@dasmaninstitute.org (I.A.); preethi.cherian@dasmaninstitute.org (P.C.); jehad.abubakr@dasmaninstitute.org (J.A.); mohamed.abufarha@dasmaninstitute.org (M.A.-F.)

**Keywords:** CD, ANGPTL8, infliximab

## Abstract

**Background:** Crohn’s disease (CD) is a chronic disease perpetuated through key pro-inflammatory molecules, including tumor necrosis factor-alpha (TNFα). Angiopoietin-like protein 8 (ANGPTL8) may contribute to inflammation cascades. This study aimed to investigate how ANGPTL8 levels are influenced in patients with CD prior to and following anti-TNF therapy. **Methods:** Patients were divided into 3 groups. Patients with CD in clinical remission receiving IFX for at least 24 weeks (IFX-experienced group), patients scheduled to start IFX (IFX-naïve group), and healthy controls (control group). In the IFX-experienced group, ANGPTL8 levels were measured 24 h before the next maintenance IFX dose. In the IFX-naïve group, levels were measured at week 0 and week 24, and in the control group, they were measured randomly. **Results:** The total number of participants was 166. The numbers of IFX-experienced, IFX-naïve patients, and healthy controls were 82, 13, and 71, respectively. Mean age ranged from 27 to 33 years of age across the three groups. Eighty-four (51%) participants were female. ANGPTL8 levels were significantly higher in patients with CD (138.26 ± 8.47 pmol) compared to the healthy control group (102.52 ± 5.99 pmol, *p* = 0.001). Among IFX-naïve patients receiving anti-TNFα treatment, ANGPTL8 levels decreased significantly from 145.06 ± 17.93 pmol pre-treatment (week 0) to 81.78 ± 10.61 pmol post-treatment (week 24), *p* = 0.007. **Conclusions:** Our findings suggest that ANGPTL8 levels are elevated in CD and may be involved in the inflammatory process. The marked reduction in ANGPTL8 levels following anti-TNFα treatment indicates its potential as a biomarker for treatment response. Further research should focus on the exact mechanisms through which ANGPTL8 influences CD progression and its utility in clinical practice.

## 1. Introduction

Crohn’s disease (CD), a key subset of inflammatory bowel disease (IBD), is characterized by chronic gastrointestinal inflammation. It has a complex pathogenesis, influenced by genetic predisposition and immune dysregulation, along with gut microbial and environmental factors, most notably cigarette smoking [1,2]. The rising global prevalence of CD, more recently in Asia and South America, warrants critical advancements in its diagnosis and treatment [2,3,4]. In recent decades, an increased understanding of the disease has led toward improved diagnostic accuracy, such as novel non-invasive imaging (magnetic resonance enterography) and biomarkers (calprotectin, lactoferrin, microRNAs, etc.) [2,5]. The management of CD is now focused on a treat-to-target approach, aiming for mucosal healing and sustained remission using non-steroidal agents to limit complications and surgical interventions. This strategy is supported by the introduction of biological therapies that target specific inflammatory mediators, such as tumour necrosis factor-alpha (TNFα), interleukin (IL)-12, and IL-23, as well as advancements in minimally invasive surgical techniques [1,2].

TNFα is a pivotal pro-inflammatory cytokine implicated in CD as well as several autoimmune diseases [2]. Elevated levels of TNFα have been observed in the intestinal mucosa, stool, and serum of patients with CD, with levels significantly correlating with disease activity [6]. In the therapeutic context, anti-TNF monoclonal antibodies have shown promising benefits in the management of CD by promoting apoptosis in activated T-cells, along with maintaining tight junction integrity in the gastrointestinal epithelium and ameliorating apoptosis [6,7].

In our previous study [8], we showed that the ANGPTL8 R59W variant was associated with increased TNFα circulation. Angiopoietin-like proteins (ANGPTLs) are a family of glycoproteins that share structural similarity with angiopoietins but lack the capacity to bind with traditional angiopoietin receptors, such as Tie1 or Tie2 [9]. They comprise eight members (ANGPTL1–8) and play significant roles in regulating lipid metabolism, inflammation, insulin resistance, and other metabolic disorders [10,11,12]. There are ongoing investigations regarding their contributions to many prevalent diseases, such as acute coronary syndrome [13]. Among the eight members, ANGPTL8, also known as betatrophin, lipasin, and RIFL (refeeding induced in fat and liver), is explored in various studies due to its role in inflammation propagation along with plasma triglyceride (TG) level regulation [14]. ANGPTL8’s interaction with TNFα and nuclear transcription factor NF-κB, a main driver in inflammatory signaling pathways through promoting pro-inflammatory gene alterations, is a subject of recent interest and investigation [15]. Due to limited data, there is growing interest in illuminating ANGTPTL8’s behavior in relation to CD disease activity, inclusive of its response to biological anti-inflammatory therapy.

In this study, we aimed to investigate the changes in ANGPTL8 levels in patients with CD compared to a healthy population. Additionally, we aimed to investigate the impact of anti-TNF therapy on ANGPTL8 levels.

## 2. Materials and Methods

### 2.1. Study Design

This is a prospective multi-center observational study and it was performed and reported in accordance with Strengthening the Reporting of Observational Studies in Epidemiology (STROBE) guidelines [16]. Adult patients with CD were recruited from multiple centers between January 2022 and June 2024.

### 2.2. Study Population and Outcome Measures

Patients were divided into 3 groups: patients with CD in clinical remission receiving IFX for at least 24 weeks for moderate to severe CD (IFX-experienced group), patients with moderate to severe CD scheduled to start IFX (IFX-naïve group), and healthy controls (control group). ANGPTL8 levels were measured 24 h before the next maintenance IFX dose in the IFX-experienced group, 24 h before starting the first dose of infliximab in the IFX-naïve group, and randomly in the control group.

An additional analysis was conducted in anti-TNF-naïve patients to assess ANGPTL8 levels before and after initiating anti-TNF therapy. ANGPTL8 levels were measured at week 0 and week 24 in the IFX-naïve group,

In this study, we specified the inclusion criteria as follows: (1) patients aged ≥ 18 years; (2) patients with moderate-to-severe Crohn’s disease [17], defined as a Crohn’s Disease Activity Index [CDAI] >220); patients with Simple Endoscopic Score for Crohn’s Disease (SES-CD) ≥ 7; (3) patients on IFX, (4) patients who have been on their current biologic therapy for at least 24 weeks, and (5) in the biologic-naïve group, patients should not have received biologic therapy.

The exclusion criteria were specified as follows: pregnant patients, pediatric patients aged <18 years, patients with missing outcome data, and patients for whom colonoscopic examination of the terminal ileum was not assessed. Patients taking concomitant corticosteroids or other immunomodulators were excluded as well.

The baseline demographic characteristics of the participants were collected retrospectively from electronic medical records (EMRs); these included age, sex, ethnicity, and body mass index (BMI). Baseline (prior to treatment) as well as post-treatment clinical, biomarker, and endoscopic data were collected from EMRs as well.

Ethical approval for this study was obtained on 3 January 2022 from the standing committee for the coordination of health and medical research at the ministry of health of Kuwait (IRB No. 2144/2022) and as per the updated guidelines of the Declaration of Helsinki (64th WMA General Assembly, Fortaleza, Brazil, October 2013) and the US Federal Policy for the Protection of Human Subjects.

### 2.3. Blood Processing

Blood samples were collected in EDTA tubes and centrifuged at 400× *g* for 10 min at room temperature to separate the plasma. The plasma was centrifuged at 800× *g* for 10 min to obtain a clear supernatant, which was aliquoted into fresh tubes. Storage specifications of the subjects’ plasma for future tests required the samples to be stored at −80 °C. For the ELISA assay, the subjects’ samples were thawed at room temperature and centrifuged at 10,000× *g* for 5 min to remove any particles or precipitates. Samples were aliquoted into the appropriate plate layout.

### 2.4. Measurement of Circulating Levels of ANGPTL8 by ELISA

Levels of human ANGPTL8 in plasma were determined using the ANGPTL8 ELISA Kit (Human) (Cat. No. 27795) from Immuno-Biological Laboratories Co. (Naka, Fujioka-Shi, Gunma, Japan). Subjects’ samples were diluted 1:20 with 1× EIA buffer from the kit, and an ELISA assay was performed following the manufacturer’s recommendations and protocol. The ELISA plate was read at 450 nm using a plate reader within 30 min of stopping the reaction. The color intensity was directly proportional to the ANGPTL8 level in the sample. The concentration of ANGPTL8 in the samples was extrapolated from the ANGPTL8 standard curve. The intra-assay and inter-assay coefficients of variation (CV%) were less than 5%.

### 2.5. Statistical Analysis

All statistical analyses were performed using SPSS for Windows, version 25.0 (IBM SPSS Inc., Chicago, IL, USA). A normality test was conducted for continuous variables, accordingly for variables with normal distribution. One-way analysis of variance (ANOVA) was used to compare the three study groups: IFX-experienced, IFX-naïve, and Healthy. To assess pairwise statistical significance, the Bonferroni post hoc test was applied for multiple comparisons. Descriptive statistics were used to summarize patient demographics and baseline characteristics for non-parametric data. Continuous variables were reported as the mean ± standard error mean (SEM), while categorical variables were presented as frequencies and percentages. The Chi-square test of independence was used to assess the differences in categorical variables across the three groups. A *p*-value of <0.05 was considered statistically significant. Finally, a generalized linear model (GLM) with a Poisson distribution and a log link function was performed to model the ANGPTL8 level based on age, BMI, gender, and corticosteroid use. The model fit the data well, as indicated by the goodness-of-fit statistics. Deviance/df = 4.493, Pearson Chi-Square/df = 5.013, AIC = 376.01. The omnibus test significant chi-square (5) = 29.843 (*p* < 0.001) indicated that the model provided a better fit to the data compared to the intercept-only model.

## 3. Results

### 3.1. Study Demographics

**Figure 1:**
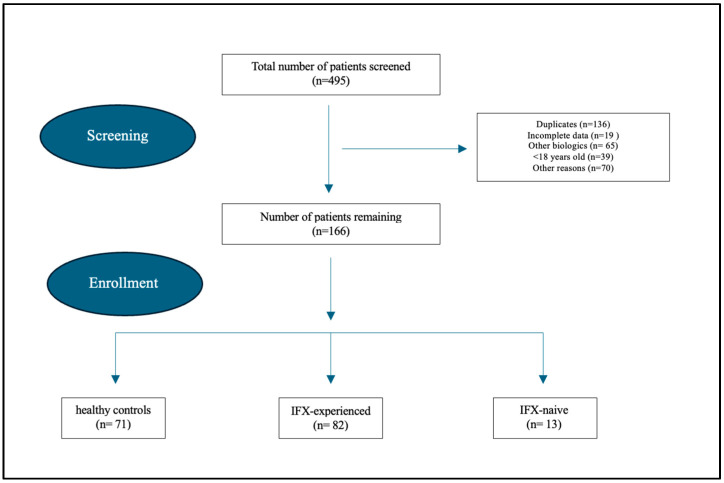
Patient enrollment process.

A total of 495 patients were screened. After applying our inclusion/exclusion criteria, 166 patients were eligible to be included in this study (Figure 1). The number of healthy controls, IFX-experienced, and IFX-naïve patients were 71, 82, and 13, respectively. Mean age ranged from 27 to 33 years of age across the three groups, and most were non-smokers [40 (24.1%)]. In total, 84 (51%) patients were females. However, in the IFX-experienced and IFX-naïve groups, the majority of patients were male (62.2% and 69.2%, respectively). The average BMI of patients with CD was 24, while healthy controls had an average BMI of 25. Interestingly, the IFX-naïve group had a higher CDAI mean score of 441 and a fecal calprotectin level of 493 compared to the IFX-experienced group (201 and 121, respectively). The healthy group had a baseline mean CDAI of 138 and fecal calprotectin level of 49. The proportion of patients on Azathioprine was 31.7% in the IFX-experienced group, while the IFX-naïve group had a bulk proportion of 76.9%. The healthy control group was not receiving any listed CD treatment (Table 1).

### 3.2. Main Outcomes

**Figure 2:**
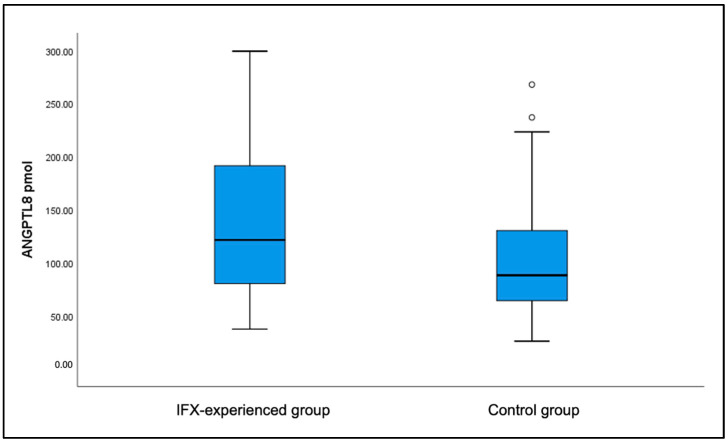
Levels of ANGPTL8 (pmol) in IFX-experienced group (135.8 ± 7.5) vs. control group (102.5 ± 6.0) (*p* = 0.001).

**Figure 3:**
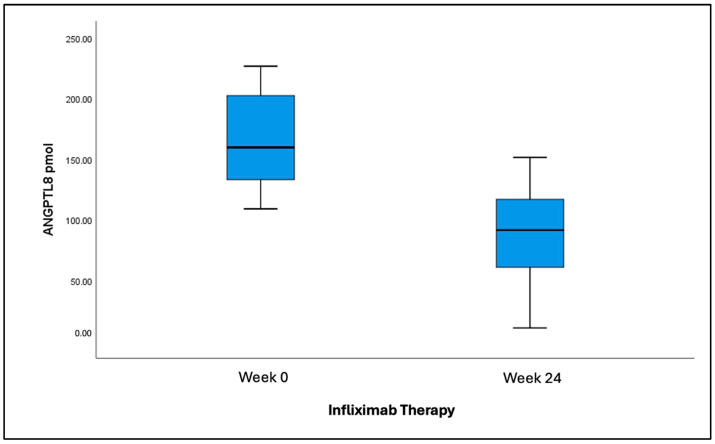
Levels of ANGPTL8 in IFX-naïve patients before (168.8 ± 10.9) vs. after therapy (85.9 ± 10.9).

ANGPTL8 levels were significantly higher in patients with CD who had already received IFX (135.8 ± 8.47 pmol) compared to the healthy control group (102.52 ± 5.99 pmol) (*p*-value < 0.001) (Figure 2). Patients in the IFX-experienced group had a lower average ANGPTL8 level of 135.8 pmol compared to patients in the IFX-naïve group, with a level of 168.8 pmol (*p*-value < 0.001). Additional analysis performed among IFX-naïve patients before and after receiving anti-TNFα treatment showed that ANGPTL8 levels decreased significantly from 145.06 ± 17.93 pmol pre-treatment (week 0) to 81.78 ± 10.61 pmol post-treatment (week 24) (*p*-value = 0.007) (Figure 3).

Regarding the individual predictors of ANGPTL8 levels, male gender (*p*-value < 0.001) and use of corticosteroids (*p*-value < 0.001) were associated with lower levels of ANGPTL8. However, age and BMI were not significant predictors for changes in ANGPTL8 levels (Table 2).

## 4. Discussion

In this study, we explored the changes in angiopoietin-like protein 8 (ANGPTL8) levels in patients with CD compared to healthy individuals and its response to anti-TNFα therapy. The findings demonstrate that circulating ANGPTL8 levels are significantly elevated in patients with CD compared to healthy controls, suggesting that ANGPTL8 may play a role in the inflammatory process, which a main characteristic of the disease. The reduction in ANGPTL8 levels following anti-TNFα treatment further supports its involvement in TNFα-driven pathways, making it a potential biomarker for monitoring therapeutic efficacy.

ANGPTL8 has been implicated in both metabolic regulation and inflammation, two processes closely linked in chronic diseases such as CD. Elevated ANGPTL8 levels have been associated with dysregulated lipid metabolism, as evidenced by its role in modulating lipoprotein lipase (LPL) activity and its involvement in triglyceride metabolism [18]. Guo et al. [19] looked at how ANGPTL8 secreted from adipose tissue is associated with inflammation and metabolic derangements. It was found that, in lipid metabolism, ANGPTL8 genes are associated with apolipoprotein C3 (APOC3) genes. There remains controversy on the subject of single nucleotide polymorphisms associated between APOC3 and ANGPTL 8 genes with regard to metabolic dysfunction-associated steatotic liver disease (MASLD) [20]. Another study [21] highlighted the link between triglyceride regulation by ANGPTL8 and its emergence as a potential novel biomarker for steatosis/steatohepatitis. This is particularly relevant in the context of CD, where metabolic disturbances often accompany chronic inflammation. In our study, the elevated levels of ANGPTL8 in patients with CD could indicate that ANGPTL8 is involved in metabolic dysregulation in addition to inflammatory signaling pathways, thus expanding the pathophysiological picture of the nature of the disease.

In addition, many previous studies showed the steady involvement of ANGPTL8 in metabolic disorders. Its levels are raised in pre-diabetes, diabetes type 1, diabetes type 2, and insulin resistance. This is due to complex interactions with glucose metabolism, triglycerides, and increases in beta cell proliferation and insulin secretion [19,20,21,22,23,24,25]. Furthermore, several studies showed ANGPTL8 associations with metabolic syndrome, dyslipidemia, and metabolic-associated fatty liver disease [26,27,28]. Interestingly, a study showed that there was a more significant increase in ANGPTL8 levels in patients with diabetic nephropathy compared to diabetic patients without the associated nephropathy [29]. Primary nephrotic syndrome showed links between ANGPTL8 and the development of hyperlipidemia and proteinuria [30].

The contemporary field examining ANGPTL8 levels and the cardiovascular system has shared some similar findings. There is interest in ANGPTL8 levels and their suitability to gauge endothelial dysfunction and grade of stenosis in coronary arteries [31]. Others aimed to see if targeting angiopoietin-like proteins can be of therapeutic benefit [32]. More studies are assessing whether there is potential for ANGPTL8 as a marker to measure long-term risk of secondary coronary events from stabilized coronary disease build-up [33,34]. Thorin et al. [32] delved into how heart structure and vessel hypertrophy interact with ANGPTL8 levels, suggesting a protective role in certain cardiac cases and other times a pathological role leading to unwanted hypertrophy [35,36].

The role of ANGPTL8 could be more complex than initially thought. Abu-Farha et al. [8] conducted a study indicating that ANGPTL8 could have pleiotropic aspects in metabolic tissues and in other organs. In chronic inflammatory disease states, it contributes to the destructive process, while on other occasions it regulates metabolism and has a defensive aspect. This seems to support the pathophysiological nature of CD, where chronic inflammation and relapses are characteristic of the nature of the disease.

ANGPTL8 has a role in metabolic and inflammatory processes, heavily interacting with glucocorticoids in a rhythmic manner whereby ANGPTL8 levels and expression oscillate and regulate lipid metabolism. A study showed that ANGPTL8 is linked to lipid metabolism involving glucocorticoids, a cause of non-alcoholic fatty liver disease in chronic exposure, and they demonstrated that ANGPTL8 levels and expression were suppressed with glucocorticoids through glucocorticoid receptors. After introducing mifepristone, an anti-glucocorticoid drug, ANGPTL8 expression and levels increased [37]. Considering that a sizable number of IFX-naïve patients had corticosteroids in their treatment regimen, it would be expected that ANGPTL8 levels would be suppressed before introducing anti-TNF therapy. The significant reduction in ANGPTL8 levels at 24 weeks could imply that the average level prior to anti-TNF therapy was higher; furthermore, the lower levels at 24 weeks suggest that there was room for a more effective treatment regimen to control CD.

ANGPTL8 is thought to be modulated by inflammatory cytokines and may act as a mediator of immunometabolic changes during active inflammatory diseases. This is supported by previous studies showing that ANGPTL8 expression increases in response to systemic inflammation [8,38]. Importantly, ANGPTL8 is linked to NAFLD, insulin resistance, and type 2 diabetes [10,39,40]. In CD, this may suggest a dual role for ANGPTL8 as both a marker of active inflammation and an indicator of metabolic comorbidity risk and could serve as a link between chronic inflammation and increased cardiometabolic risk. Moreover, in our cohort, ANGPTL8 levels decreased significantly following anti-TNFα therapy in IFX-naïve patients (*p* = 0.007), suggesting its potential as a prognostic biomarker for treatment response. This aligns with prior work showing that biologic therapies can reduce systemic inflammation and improve insulin sensitivity, hence the reduction in ANGPTL8 levels [41].

Zhang et al. [38] proposed a molecular mechanism by which TNFα regulates ANGPTL8. Activation of nuclear factor-κB (NF-κB) is mediated by TNFα, leading to excessive inflammation. Authors demonstrated that ANGPTL8 functions as a negative feedback regulator in TNFα-triggered cellular NF-κB activation. Additionally, Abu-Farha et al. [8] indicated that ANGPTL8 can act as a co-receptor of p62 in selective autophagy, and this interaction is necessary for the degradation of IKKγ, a key regulator of NF-κB. Furthermore, ANGPTL8 has been implicated in enhancing NF-κB signaling, particularly in conditions of TNFα stimulation where NF-κB inhibitor was phosphorylated at a more rapid pace. The reduction in ANGPTL8 levels after anti-TNFα therapy suggests that targeting TNFα not only suppresses NF-κB activation but may also reduce ANGPTL8 levels through mechanisms that are not fully understood.

The significant reduction in ANGPTL8 levels observed after anti-TNFα therapy suggests that ANGPTL8 could potentially serve as a biomarker for therapeutic response in CD patients. Traditional biomarkers such as C-reactive protein (CRP) and fecal calprotectin are commonly used to monitor inflammation in CD. However, ANGPTL8 could complement these markers by providing additional information about both metabolic and inflammatory status. As observed in our study, ANGPTL8 levels were significantly elevated in patients with CD compared to healthy controls, and ANGPTL8 levels decreased following anti-TNFα treatment. However, it is important to consider the biological plausibility and clinical utility of these changes and whether the observed difference is sufficient to guide clinical decision making or track treatment response. Further research is needed to validate ANGPTL8’s utility in clinical practice, but the findings from this study offer promising evidence for its role as a biomarker in CD management.

The notable strengths of this study are that it is the first study, to our knowledge, to describe an association between CD, anti-TNF therapy, and ANGPTL8, and one of the few studies investigating the effect of this protein on inflammation. Additionally, it is a well-designed observational study that controlled for several confounders, such as pregnancy, which can have massive physiological metabolic changes.

This study is not without limitations. One such limitation is that the number and location of subjects enlisted in the study could possibly lead to sampling bias. However, to mitigate that, we conducted the study prospectively in multiple tertiary centers with an extended length of the enrolment process to try to capture as many subjects as possible. Additionally, no randomization or matching was employed during participant recruitment in our study; however, we performed a generalized linear model (GLM) with a Poisson distribution and a log link function to investigate variations in ANGPTL8 levels based on age, BMI, gender, and corticosteroid use. Although ANGPTL8 levels showed some inter-individual variability, this is expected given known influences such as BMI, metabolic status, and insulin resistance [42,43,44]. Despite this, significant group differences and consistent trends indicate that ANGPTL8 changes are biologically meaningful and reproducible.

## 5. Conclusions

The elevated levels of ANGPTL8 in patients with CD and their significant reduction following anti-TNFα therapy highlight its potential involvement in the inflammatory process. Future studies should explore the therapeutic potential of targeting ANGPTL8 in combination with anti-TNFα therapies, such as how rapidly it responds to anti-TNFα therapy and target level reduction in relation to disease activity.

## Figures and Tables

**Table 1:** Demographic characteristics of all included participants.

Variables	IFX-Experienced (n = 82)	IFX-Naïve Week 0	Healthy	ǂ *p*-Value	Multiple Comparisons with Post Hoc Bonferroni (*p*-Value)		
		(n = 13)	(n = 71)		IFX-Experienced vs. IFX-Naïve	IFX-Experienced vs. Healthy	IFX-Naïve vs. Healthy
Age, mean ± SEM	29.76 ± 1.4	27.92 ± 3.82	33.66 ± 1.55	0.113	0.104	0.051	0.063
BMI	24.22 ± 0.60	24.06 ± 1.31	29.53 ± 0.83	<0.001	1.000	<0.001	<0.001
Male	51 (62.2%)	9 (69.2%)	22 (31.0%)	<0.001			
Female	31 (37.8%)	4 (30.8%)	49 (69.0%)				
Smoking	23 (28.0%)	4 (30.8%)	13 (18.3%)	0.308			
CDAI score, mean ± SD	201 ± 11	441 ± 12	138 ± 7	<0.001	<0.001	<0.001	<0.001
Fecal Cal, μg/mg	121	493	49				
CRP, mg/L	12.3	31	7				
Albumin, g/L	49	55	33				
Azathioprine	26 (31.7%)	10 (76.9%)	0 (0.0)				
Methotrexate	4 (4.9%)	0 (0.0)	0 (0.0)				
Corticosteroids	5 (6.1%)	5 (38.5%)	0 (0.0)				
ANGPTL8, pmol	135.8 ± 7.5	168.8 ± 10.9	102.5 ± 6.0	<0.001	<0.001	<0.001	<0.001

ǂ One-way ANOVA *p*-value, ANGPTL8; Angiopoietin-like protein 8, BMI: Body mass index, CDAI: Crohn’s Disease Activity Index, CRP: C-reactive protein, Fecal Cal: Fecal calprotectin, SEM: Standard error of the mean.

**Table 2:** Generalized linear model (Poisson with log link) predicting ANGPTL8 as a biomarker.

Predictor	Beta	SE	Wald χ²	*p*-Value	95% CI for Beta
Intercept	4.824	0.064	27.88	<0.001	[3.699–4.949]
Age	0.032	0.001	2.039	0.153	[0.020–0.047]
BMI	0.024	0.002	5.010	0.025 *	[0.011–0.043]
Gender (Male vs. Female)	−0.173	0.020	9.600	<0.001 *	[−0.212–−0.135]
Use of Corticosteroid (Yes vs. No)	−0.229	0.040	6.924	<0.001 *	[−0.308–−0.150]

Dependent Variable: ANGTPL8; SE: Standard Error; * Significant *p*-value.

## Data Availability

The data presented in this study are available upon request from the corresponding author due to ethical restrictions.

## Abbreviations

The following abbreviations are used in this manuscript:

ANGPTL8	Angiopoietin-like protein 8
Anti-TNFα	Tumor necrosis factor antagonist
CD	Crohn’s disease
CRP	C-reactive protein
IFX	Infliximab
